# Factors Associated with Pressure Injury Occurrence in Older Trauma Patients

**DOI:** 10.3390/healthcare14010100

**Published:** 2025-12-31

**Authors:** Minjun Kim, Seunghye Choi

**Affiliations:** 1 Department of Trauma Surgery, Incheon Regional Trauma Center, Gachon University Gil Medical Center, 21 Namdong-daero, Yeonsu-gu, Incheon 21565, Republic of Korea; 1@gilhospital.com; 2 College of Nursing, Gachon University, 191 Hambakmoero, Yeonsu-gu, Incheon 21936, Republic of Korea

**Keywords:** older patients, elderly, trauma, pressure injury, frailty

## Abstract

**Background/Objectives:** Older individuals are more vulnerable to stress and trauma. Although pressure injuries (PIs) are recognized as a significant complication, the specific impact of frailty on PI development in older Asian trauma patients remains insufficiently explored. This study aims to investigate the factors associated with the occurrence of hospital-acquired pressure injuries (HAPU) in older patients aged ≥65 years, including frailty. **Methods:** This study is a retrospective secondary data analysis of 3418 older trauma patients admitted to a regional trauma center (including ICU and trauma ward) from 1 January 2020 to 31 December 2023. Patients with PIs present on admission (POA) were excluded to strictly analyze new PI occurrence. Frailty was assessed using the mFI-5. **Results:** The mean age of participants was 77.33 years. During hospitalization, 2.5% (*n* = 84) of patients developed new PIs. Multivariate logistic regression identified that higher frailty score (Odds Ratio [OR] = 1.59, 95% Confidence Interval [CI]: 1.26–2.02), lower BMI (OR = 0.93, 95% CI: 0.86–0.99), hypoalbuminemia (OR = 0.55, 95% CI: 0.36–0.84), and prolonged hospital stay (OR = 1.05, 95% CI: 1.04–1.06) were independently associated with PI occurrence. Chronological age was not a significant predictor in the multivariate model. **Conclusions:** Frailty, nutritional status (BMI, albumin), and prolonged hospital stay are significant factors associated with HAPU in older trauma patients.

## 1. Introduction

Older individuals exhibit frailty, fatigue, weight loss, decreased balance, reduced activity, slowed performance, social withdrawal, mild cognitive changes, and higher vulnerability to stressors [1]; consequently, the incidence of trauma among older people is gradually increasing [2]. In Korea, individuals aged ≥60 years constitute 51.3% of all severe trauma patients, with those in their 60s accounting for 23.2%, those in their 70s for 16.1%, and those aged ≥80 years for 12.0% [3]. The main mechanisms of injury for Korean older individuals are falls and slips, followed by traffic accidents (TAs), collisions, poisonings, and lacerations [4]. Furthermore, compared to the average length of stay (LOS) of 13 days for all patients with injury, the average LOS for older patients with injury was longer at 16 days [4].

The prognosis of older trauma patients is known to be attributed to numerous factors, including not only the Injury Severity Score (ISS) but also age-related physiological changes associated with frailty, comorbidities, malnutrition, and a decline in the functional status [5]. Frailty increases with age, is more prevalent in women than in men, is more common in individuals with chronic diseases [6], and increases the incidence of trauma, such as falls and fractures [7]. Due to the underlying diseases, older trauma patients have a higher likelihood of developing complications during treatment and recovery, showing higher complication rates compared to younger trauma patients [8]. Among older patients, it has been reported that the risk of mortality is significantly increased in those who develop a pressure injury (PI) as a complication compared to those who do not [9].

One of the important prognostic factors in trauma in older patients is the injured body region; mortality was reported to be significantly higher in cases of head and neck injuries, while injuries to the extremities and pelvis were often associated with changes in the functional status even after discharge [10]. According to a previous study, 2.5% of older trauma patients with hip fractures developed PIs perioperatively [11]. Perioperative PIs occurred more frequently in older male patients, and the group with PIs exhibited a 2.5-fold increase in the 1-year mortality compared to the group without PI [11].

According to a previous study, the risk factors for PI in patients are diverse and include patient characteristics, admission route, LOS, intrinsic factors (e.g., level of consciousness, nutritional status), use of medical devices, and use of vasopressors [12]. Older trauma patients in the Emergency Department (ED) are in an environment where they are susceptible to PIs induced by extrinsic factors such as backboards, ED stretchers, and MRI or computed tomography scan tables; therefore, it is important to manage preventable PIs from the initial stage of hospitalization [13].

Therefore, comprehensive assessment guidelines are needed. While previous studies have identified general risk factors, the specific impact of frailty (measured by mFI-5) in the context of Asian older trauma patients has not been sufficiently explored compared to Western populations. Furthermore, distinguishing the independent effect of frailty from chronological age requires detailed investigation. This study aimed to investigate the factors associated with the development of hospital-acquired PIs in older trauma patients aged ≥65 years, utilizing the mFI-5 to assess physiological reserve beyond simple age.

## 2. Materials and Methods

### 2.1. Study Design

This study is a retrospective secondary data analysis utilizing data merged from the Korea Trauma Data Bank (KTDB) registry and Electronic Medical Records (EMR).

### 2.2. Participants

This study utilized the medical records and Korea Trauma Data Bank (KTDB) data of older trauma patients (*n* = 3468) aged ≥65 years who were admitted through a single tertiary hospital’s regional trauma center in Incheon over a 4-year period from 1 January 2020 to 31 December 2023. The KTDB is a national trauma data entry program that collects information related to trauma patients transmitted from regional trauma centers to establish the foundation for the trauma care system and provide basic data for trauma-related research and policy-making by collecting. Excluding patients with an unclear history of trauma (*n* = 3), patients who were dead on arrival (DOA; *n* = 3), and patients with missing critical information for analysis (*n* = 44), a total of 3418 individuals were included in the final analysis. Specifically, patients identified with pressure injuries present on admission (POA) during the initial skin assessment were excluded from the study population to ensure that the analysis focused solely on hospital-acquired pressure injuries (HAPU). Patients were repositioned every 2 h unless contraindicated by spinal instability and used air mattresses according to the institution’s guidelines for PIs prevention. In 2023, new sections were added regarding PIs related to medical devices and the surgical management of PIs [14].

### 2.3. Instruments

The investigated items were collected and categorized into general characteristics (sex, age, body mass index [BMI], presence of cohabiting family, presence of comorbidities, smoking, alcohol consumption, employment status), frailty, and clinical characteristics.

#### 2.3.1. BMI

In this study, BMI was calculated as kg/m^2^ based on height (meter) and weight (kg) from medical records [15]. BMI cutoffs were categorized according to the criteria for Asian and Pacific populations as underweight (<18.5 kg/m^2^), normal weight (18.5–22.9 kg/m^2^), overweight (23–24.9 kg/m^2^), and obese (≥25 kg/m^2^) [16]. In the logistic regression model, BMI was analyzed as a continuous variable to capture the linear risk associated with each unit decrease, preventing information loss from categorization

#### 2.3.2. Modified Frailty Index (mFI-5)

In this study, the frailty of older patients admitted for trauma was assessed using the mFI-5. The mFI-5 assesses the following items: diabetes mellitus (DM), hypertension requiring medication, chronic obstructive pulmonary disease (COPD), congestive heart failure (CHF), and partially dependent functional status, with 1 point assigned for each applicable item (ranging from 0 to 5) [17]. A higher total score across the five items signifies an increased state of frailty [17]. The “partially dependent functional status” was determined based on the patient’s pre-injury baseline status obtained during history taking at admission, not the acute functional decline caused by the trauma.

#### 2.3.3. Clinical Characteristics

Clinical characteristics included the type of accident, initially measured systolic blood pressure (SBP), diastolic blood pressure (DBP), mean arterial blood pressure (mABP), body temperature (BT), pulse rate (PR), serum albumin level (g/dL), Glasgow Coma Scale (GCS), weighted Revised Trauma Score (RTS), the most severely injured body region, Injury Severity Score (ISS), LOS, clinical outcome (death/survival), and the development of new PIs during hospitalization. The “occurrence” strictly refers to new PIs developed during the hospital stay. A serum albumin level of ≤3.5 g/dL was defined as hypoalbuminemia [18].

The type of accident was classified as TA (all transport accidents caused by a means of transport used to carry people or goods), falls (falling or being pushed from a higher level, slipping or rolling on stairs, jumping, etc.), slips (slipping or tripping on the same level with feet on the ground), and other accidents [19].

The GCS is calculated from a minimum of 3 points (worst) to a maximum of 15 points (best), where GCS scores of 3–8, 9–12, and 13–15 indicate severe, moderate, and mild head injuries, respectively [20].

The weighted RTS is the sum of the patient’s GCS, systolic blood pressure, and respiratory rate scores upon admission, each multiplied by its respective constant. It ranges from a minimum of 0 to a maximum of 7.8408, where a higher score indicates a higher probability of survival, and patients with a score below 4 require treatment at a specialized trauma center [21].

The most severely injured body region refers to the most injured region among the following body divisions: head/neck, face, thorax, abdomen, extremity/pelvis, and external [22]. In this study, it refers to the body region with the highest Abbreviated Injury Scale (AIS) score, which presents the severity of injury in six grades from 1 to 6 based on the degree of risk to life (Association for the Advancement of Automotive Medicine, 1990) [23].

The ISS is a method for assessing the severity of multiple injuries by scoring them. The body is divided into six regions (head/neck, face, thorax, abdomen, extremities, external surface), each injury is scored by severity with the AIS, and the ISS is calculated as the sum of the squares of the highest AIS scores in the three most severely injured body regions [24]. ISS = (1st AIS score)^2^ + (2nd AIS score)^2^ + (3rd AIS score)^2^.

It is known that mortality increases sharply with an ISS of 16 or higher, and generally, scores of 1–8 are defined as mild trauma, 9–15 as moderate trauma, 16–24 as severe trauma, and ≥25 as very severe trauma [25].

PIs were assessed by trained nurses upon admission and daily thereafter using the NPUAP (National Pressure Ulcer Advisory Panel) staging system using the Braden Scale [26]. The assessment included skin inspection of bony prominences. Only new PIs that developed during the hospital stay (HAPU) were classified as “occurrence.” Stage 1 to Stage 4 and unstageable injuries [26] were all included in the event group.

### 2.4. Ethical Consideration

Prior to collecting the original dataset, permission for data use was obtained from the director of the regional trauma center at G Hospital in Incheon after explaining the purpose of this study. Subsequently, this study was approved by the Institutional Review Board (IRB) of G University (IRB No. 1044396-202501-HR-016-01) with a waiver of informed consent due to the retrospective nature of this study.

### 2.5. Data Analysis

Data were analyzed using the IBM SPSS version 26. The threshold for statistical significance was set at *p* < 0.05 for all the analyses. The general and clinical characteristics of the participants were calculated using the real number, percentage, mean and standard deviation. Missing data were handled using complete-case analysis; patients with missing values for key predictors were excluded from the regression model. A *t*-test and χ^2^-test were conducted to identify the differences between the general and clinical characteristics of patients according to the PI.

A multiple logistic regression analysis was conducted to examine the factors associated with the PI. The multiple logistic regression analysis included variables with significant differences in PI by univariate analysis. Prior to performing the multiple logistic regression, the Hosmer and Lemeshow test was conducted to identify the goodness of fit. Multicollinearity was checked using Variance Inflation Factors (VIF), and model discrimination was assessed using the C-statistic (Area Under the Curve, AUC).

## 3. Results

### 3.1. General Characteristics, and Frailty of the Participants

The average age of the participants was 77.33 (SD = 8.04). Among them, 45.4% (*n* = 1553) were male, 68.8% (*n* = 2352) had cohabiting family, 12.9% (*n* = 441) were smokers, 22.1% (*n* = 757) consumed alcohol, 22.4% (*n* = 765) were employed, and 96.2% (*n* = 2901) had comorbidities. Among the older trauma patients, 16.1% (*n* = 552) scored 0 on the frailty assessment tool, 33.3% (*n* = 1139) scored 1, 32.2% (*n* = 1102) scored 2, and 18.3% (*n* = 625) scored 3 or higher (Table 1).

### 3.2. Clinical Characteristics of the Participants

The most common type of accident causing trauma was slips (54.7%, *n* = 1869), followed by TAs and falls. The proportion of participants with hypoalbuminemia was 15.9% (*n* = 513). Upon admission, GCS assessment classified 0.4% (*n* = 15) as severe, 1.8% (*n* = 62) as moderate, and 85.1% (*n* = 2910) as mild. Regarding GCS, 12.6% were “Unable to assess” due to intubation/sedation; these patients were included in the multivariate analysis using the Weighted RTS and ISS, which account for physiological severity.

The most common primary injury location was the extremity/pelvis (41.5%, *n* = 1417). The mean weighted RTS score was 7.68 ± 0.59. Based on the ISS, 26% (*n* = 887) were classified as mild, 50.1% (*n* = 1711) as moderate, 13.5% (*n* = 460) as severe, and 10.5% (*n* = 360) as very severe. After admission, 7.5% (*n* = 256) of the participants died, and 92.5% (*n* = 3162) survived. The average LOS was 14.31 days (SD = 13.03). During hospitalization, 2.5% (*n* = 84) of the participants developed a PI (Table 2).

### 3.3. PI Occurrence According to General Characteristics and Frailty

Univariate analysis revealed that the PI group had significantly higher age (*t* = 2.29, *p* = 0.011) and frailty score (*t* = 5.312, *p* < 0.001), and a significantly lower BMI (*t* = −2.547, *p* = 0.005) compared to the non-PI group (Table 3).

### 3.4. PI Occurrence According to Clinical Characteristics

Univariate analysis demonstrated that the PI group had significantly higher PR (*t* = 2.291, *p* = 0.012), ISS (*t* = 2.690, *p* = 0.004), and LOS (*t* = 8.271, *p* < 0.001), and significantly lower mABP (*t* = −1.905, *p* = 0.028) and lower albumin level (*t* = −5.026, *p* < 0.001) compared to the non-PI group (Table 4). The mean LOS for all subjects was 14.31 days (Table 2), whereas the PI group had a significantly longer mean LOS of 35.37 days.

### 3.5. Factors Associated with PI Occurrence

Prior to the logistic regression analyses, the Hosmer-Lemeshow test was conducted to confirm the goodness of fit of the model. The results showed that the model was suitable (χ^2^ test = 7.745, *p* = 0.459). The model demonstrated acceptable discrimination with a C-statistic (AUC) of 0.86. VIF values for all predictors were below 2.5, indicating no significant multicollinearity. Moreover, we examined the standardized residuals from the regression analysis and confirmed that continuous variables are close to the diagonal line, forming a straight-line pattern.

In the logistic regression model (enter mode), the variables age, BMI, mFI-5, mABP, PR, albumin, ISS, LOS were analyzed. Logistic regression analysis showed that low BMI, high frailty, low albumin level, and long LOS were independently associated with PI onset (R^2^ = 19.1%, *p* = 0.026, *p* < 0.001, *p* = 0.005, *p* < 0.001, respectively; Table 5). Notably, while age was significant in univariate analysis, it was not significant in the multivariate model (*p* = 0.133), whereas mFI-5 remained highly significant (*p* < 0.001).

## 4. Discussion

This study attempted to closely investigate the characteristics of older trauma patients aged ≥65 years admitted through a single tertiary hospital’s regional trauma center and to investigate the factors influencing the development of PIs, including the degree of frailty. The results of this study indicated that the mortality rate of older trauma patients was 7.5%, and the incidence of PI during hospitalization was 2.5%. Although this study did not investigate the timing of PI onset to determine if it occurred in the ED, a previous study reported that 5.2% of subjects who arrived at the ED by ambulance developed a PI during their ED stay, and 7.8% developed one during their total hospital stay [26]. For older individuals, the development of a PI can be a factor that increases mortality [8], and since a PI can develop even over a short period, a thorough screening for PI development is important starting from the ED [27].

In this study, an investigation into the causes of trauma in older patients revealed that slips were the most common, followed by TA. This is similar to a previous result wherein falls and slips were determined as the main mechanisms of injury in the older Korean population [28]; however, the proportion of TA among the elderly was also high in this study.

The most common primary injury location was the extremity/pelvis, accounting for 41.5% of all participants. A previous study reported that 2.5% of older adults with a hip fracture developed a PI perioperatively, and those who developed a PI had a 2.5-fold higher 1-year mortality compared to those who did not [29]. In this study, there was a tendency toward higher mortality in the PI group (11.9% vs. 7.4%, *p* = 0.137). However, this difference did not reach statistical significance, potentially due to the limited number of PI cases (*n* = 84). Therefore, further large-scale studies involving a greater number of patients are warranted to clarify the relationship between PI occurrence and mortality. TAs were mainly associated with severe trauma; therefore, it is necessary to raise awareness about TA and develop proactive prevention strategies for older people [30].

The results of this study indicated that the factors influencing the development of pressure injuries included BMI, frailty, albumin level, and LOS. An interesting finding is the “Age Paradox,” where chronological age lost its significance in the multivariate model, while frailty (mFI-5) remained a strong predictor. This suggests that physiological reserve and functional status are more accurate determinants of tissue viability and recovery than age alone. This supports the shift from age-based to frailty-based risk stratification in trauma care. In this study, the average LOS for elderly trauma patients was 14.31 days. However, the average LOS for the PI group was 35.37 days, which was significantly longer than that of the non-PI group. According to a report by the Korea Disease Control and Prevention Agency, the proportion of injury patients among all inpatients in 2022 was 15.4%, ranking first among disease groups [29]. Furthermore, the average LOS for injury patients was 13 days, and since the LOS increases with age, injury prevention and complication management in older patients are important [29].

On the other hand, the participants’ ISS was not a factor that significantly influenced the development of PIs, probably because a high ISS is closely related to early mortality, resulting in a relatively shorter LOS for severely ill patients [31]. In this present study, the comparison of the median ISS between the non-survivor and survivor groups revealed that the median ISS for the non-survivor group was 25, significantly higher than the median ISS of 9 for the survivor group. The results of this study indicated that low BMI and hypoalbuminemia were factors influencing the development of PIs. Low BMI and hypoalbuminemia are well-known risk factors for the development of PIs [32,33]. Individuals with a low BMI are reported to have a higher risk of developing PI compared to those with a higher BMI, as the tissues around bony prominences are more susceptible to tension and stretching caused by external pressure, leading to an increased likelihood of ischemia and necrosis [34]. Hypoalbuminemia is reported as an independent risk factor for PI because it causes fluid to shift from the intravascular space to subcutaneous tissues, making the area more vulnerable to pressure injury [35]. However, there are also reports stating that hypoalbuminemia, as a biomarker, has a relatively lower predictive effect for the early occurrence of PI compared to hemoglobin [36]. In this study, Hb was not found to be a significant factor associated with PI occurrence, and further research is needed to determine whether this is attributable to the characteristics of trauma patients. The development of PIs can negatively impact the clinical outcomes of older trauma patients. Therefore, we recommend implementing proactive prevention strategies starting from the ED. This includes routine screening of mFI-5 and albumin levels upon admission. For patients identified as high-risk (high frailty, low BMI), a multidisciplinary approach involving early nutritional support teams and the immediate application of pressure-redistributing surfaces is crucial, regardless of the patient’s chronological age.

Although various indicators for predicting PI development have been established, there is a need for more proactive prevention strategies for frail older patients with low BMI and albumin levels upon ED admission.

This study has several limitations. First, as a retrospective study, causal relationships, particularly between LOS and PI, should be interpreted with caution due to potential reverse causality (i.e., PIs extending LOS). Second, detailed data on PI staging (e.g., Stage 1 vs. 4), specific nursing interventions (e.g., repositioning frequency) and detailed characteristics about subjects were not available in the registry. Third, the relatively low incidence of PI (2.5%) compared to other studies may be due to the inclusion of mild trauma patients (ISS < 9) in our denominator. Finally, albumin levels may have been influenced by the acute phase response to trauma.

## 5. Conclusions

In conclusion, this study demonstrates that frailty (mFI-5), nutritional status (BMI, Albumin), and length of stay are significant factors associated with the development of hospital-acquired pressure injuries in older trauma patients. Significantly, frailty was a superior predictor compared to chronological age. These findings underscore the need for an integrated assessment protocol that combines frailty and nutritional screening immediately upon presentation to the emergency department. Future prospective, multi-center studies are warranted to validate these findings and to evaluate the effectiveness of frailty-targeted prevention bundles.

## Figures and Tables

**Table 1:** General characteristics, and frailty of the participants (*n* = 3418).

Characteristics	Category		*N* (%) or Mean ± SD	Range
Sex	Male		1553 (45.4)	
	Female		1865 (54.6)	
Age			77.33 ± 8.04	65–105
	65–74		1382 (40.4)	
	75–84		1294 (37.9)	
	85 years and older		742 (21.7)	
BMI			23.13 ± 3.58	10.1–42.1
	Underweight	<18.5	302 (8.8)	
	Normal	18.5–22.9	1424 (41.7)	
	Overweight	23–24.9	727 (21.3)	
	Obese	≥25	965 (28.2)	
Cohabiting family	Yes		2352 (68.8)	
	No		1065 (31.2)	
Comorbidities	Yes		2901 (96.2)	
	No		115 (3.8)	
Smoking	Yes		441 (12.9)	
	No		2977 (87.1)	
Alcohol consumption	Yes		757 (22.1)	
	No		2661 (77.9)	
Employment	Yes		765 (22.4)	
	No		2653 (77.6)	
mFI-5			1.55 ± 1.01	0–5
	0		552 (16.1)	
	1		1139 (33.3)	
	2		1102 (32.2)	
	≥3		625 (18.3)	

SD, standard deviation; BMI, body mass index; mFI-5: Modified Frailty Index-5.

**Table 2:** Clinical characteristics of the participants (*n* = 3418).

Characteristics	Category		*N* (%) or Mean ± SD	Range
Type of accident	TA		670 (19.6)	
	Fall down		585 (17.1)	
	Slip down		1869 (54.7)	
	Others		294 (8.6)	
SBP			152.52 ± 32.14	40–284
DBP			86.87 ± 20.01	21–239
mBP			102.21 ± 21.54	25–223
BT			36.65 ± 0.67	34–41
PR			83.73 ± 16.99	40–200
Albumin			3.90 ± 0.53	0.90–5.20
	Normal		2718 (84.1)	
	Hypoalbuminemia		513 (15.9)	
GCS	Severe	3–8 points	15 (0.4)	
	Moderate	9–12 points	62 (1.8)	
	Mild	13–15 points	2910 (85.1)	
	Unable to assess *		431 (12.6)	
Weighted RTS			7.68 ± 0.59	2.63–7.84
Main damaged body region	Head/neck		867 (25.4)	
	Face		52 (1.5)	
	Thorax		519 (15.2)	
	Abdomen		284 (8.3)	
	Extremity/pelvis		1417 (41.5)	
	External		2 (0.1)	
	Multiple		275 (8.1)	
ISS			11.42 ± 8.02	0–75
	0–8		887 (26.0)	
	9–15		1711 (50.1)	
	16–24		460 (13.5)	
	Over 25		360 (10.5)	
LOS			14.31 ± 13.03	1–200
Clinical outcome	Death		256 (7.5)	
	Survival		3162 (92.5)	
PI	Yes		84 (2.5)	
	No		3334 (97.5)	

SD, standard deviation; TA, traffic accident; SBP, systolic blood pressure; DBP, diastolic blood pressure; mBP, mean blood pressure; BT, body temperature; PR, pulse rate; GCS, Glasgow Coma Scale; *, unable to measure due to the administration of sedatives, endotracheal intubation, etc.; RTS, Revised Trauma Score; ISS, Injury Severity Score; LOS, length of stay; PI, pressure injury.

**Table 3:** Pressure injury occurrence according to general characteristics, frailty.

		PI Group (*N* = 84)	Non-PI Group (*N* = 3334)	χ2 or *t*	Mean Difference	95% CI (Lower, Upper)	*p*-Value
		*N* (%) or Mean ± SD	*N* (%) or Mean ± SD				
Sex	Male	46 (54.8)	1507 (45.2)	3.021			0.082
	Female	38 (45.2)	1827 (54.8)				
Age		79.31 ± 7.64	77.28 ± 8.04	2.29	2.032	0.293–3.772	0.011
BMI		22.15 ± 3.69	23.15 ± 3.58	−2.547	−1.007	−1.783, −0.232	0.005
Comorbidities	Yes	80 (95.2)	3203 (96.1)	0.150			0.699
	No	4 (4.8)	131 (3.9)				
Cohabiting family	Yes	52 (61.9)	2301 (69.0)	1.932			0.165
	No	32 (38.1)	1033 (31.0)				
Smoking	Yes	14 (16.7)	427 (12.8)	1.086			0.297
	No	70 (83.3)	2907 (87.2)				
Alcohol consumption	Yes	16 (19.0)	741 (22.2)	0.480			0.488
	No	68 (81.0)	2593 (77.8)				
Employment	Yes	14 (16.7)	751 (22.5)	1.619			0.203
	No	70 (83.3)	2583 (77.5)				
mFI-5		2.12 ± 0.96	1.53 ± 1.00	5.312	0.588	0.371–0.806	<0.001

PI, pressure injury; SD, standard deviation; BMI, body mass index; mFI-5: Modified Frailty Index-5; CI, confidence interval.

**Table 4:** Pressure injury occurrence according to the clinical characteristics.

		Pressure Injury Group (*N* = 84)	Non-Pressure Injury Group (*N* = 3334)	χ^2^ or *t*	Mean Difference	95% CI (Lower, Upper)	*p*-Value
		*N* (%) or Mean ± SD	*N* (%) or Mean ± SD				
Type of accident	TA	17 (20.2)	653 (19.6)	1.066			0.785
	Fall down	13 (15.5)	572 (17.2)				
	Slip down	49 (58.3)	1820 (54.6)				
	Others	5 (6.0)	289 (8.7)				
SBP		149.76 ± 35.70	152.59 ± 32.05	−0.788	−2.831	−9.876, 4.215	0.215
DBP		84.70 ± 26.06	86.93 ± 19.83	−0.999	−2.234	−6.618, 2.151	0.159
mABP		97.80 ± 20.91	102.33 ± 21.55	−1.905	−4.531	−9.195, 0.133	0.028
BT		36.61 ± 0.85	36.65 ± 0.67	−0.535	−0.040	−0.186, 0.106	0.296
PR		88.43 ± 18.97	83.62 ± 16.92	2.291	4.817	1.119, 8.516	0.012
Albumin		3.61 ± 0.52	3.90 ± 0.53	−5.026			<0.001
	Normal	58 (69.0)	2660 (84.5)	14.674			<0.001
	Hypoalbuminemia	26 (31.0)	487 (15.5)				
GCS		14.85 ± 0.48	14.80 ± 0.88	−0.468	0.053	−0.168, 0.274	0.320
Weighted RTS		7.59 ± 0.78	7.69 ± 0.59	−1.026	−0.094	−0.278, 0.089	0.154
Main damaged body region	Head/neck	23 (27.4)	844 (25.3)	2.278			0.892
	Face	0 (0.0)	51 (1.5)				
	Thorax	13 (15.5)	506 (15.2)				
	Abdomen	5 (6.0)	279 (8.4)				
	Extremity/pelvis	37 (44.0)	1380 (41.4)				
	External	0 (0.0)	2 (0.1)				
	Multiple	6 (7.1)	269 (8.1)				
ISS		13.88 ± 8.48	11.36 ± 7.99	2.690	2.518	0.658, 4.379	0.004
LOS		35.37 ± 23.84	13.78 ± 12.18	8.271	21.59	18.86, 24.32	<0.001
Clinical outcome	Death	10 (11.9)	246 (7.4)	2.423			0.137
	Survival	74 (88.1)	3088 (92.6)				

SD, standard deviation; TA, traffic accident; SBP, systolic blood pressure; DBP, diastolic blood pressure; mABP, mean arterial blood pressure; BT, body temperature; PR, pulse rate; GCS, Glasgow Coma Scale; RTS, Revised Trauma Score; ISS, Injury Severity Score; LOS, length of stay; CI, confidence interval.

**Table 5:** Factors associated with pressure injury occurrence among older trauma patients (*n* = 3418).

Factor	B	SE	Odds Ratio	95% CI		*p*
				Lower	Upper	
Age	0.024	0.016	1.024	0.993	1.056	0.133
BMI	−0.078	0.035	0.925	0.863	0.991	0.026
mFI-5	0.466	0.121	1.594	1.258	2.019	<0.001
mABP	0.006	0.006	1.006	0.994	1.017	0.330
PR	0.010	0.006	1.011	0.999	1.023	0.084
Albumin	−0.597	0.213	0.551	0.362	0.837	0.005
ISS	−0.007	0.016	0.993	0.962	1.026	0.687
LOS	0.048	0.006	1.049	1.038	1.061	<0.001
Nagelkerke R^2^ = 19.1						

SE, standard error; CI, confidence interval; BMI, body mass index; mFI-5, Modified Frailty Index-5; mABP, mean arterial blood pressure; PR, pulse rate; ISS, Injury Severity Score; LOS, length of stay. *Note: Odds Ratios for continuous variables (Age, BMI, LOS) represent the change in risk per 1-unit increase.*

## Data Availability

The data are not publicly available due to ethical restrictions. The data presented in this study are available on request from the corresponding author.

## Abbreviations

The following abbreviations are used in this manuscript:

PI	Pressure Injury
KTDB	Korea Trauma Data Bank
mFI	Modified Frailty Index
GCS	Glasgow Coma Scale
RTS	Revised Trauma Score
ISS	Injury Severity Score
LOS	Length of Stay
AIS	Abbreviated Injury Scale
